# Synthesis of Xylitan Derivatives and Preliminary Evaluation of in Vitro Trypanocidal Activity

**DOI:** 10.3390/molecules21101342

**Published:** 2016-10-10

**Authors:** Paula Regina Elias, Gleicekelly Silva Coelho, Viviane Flores Xavier, Policarpo Ademar Sales Junior, Alvaro José Romanha, Silvane Maria Fonseca Murta, Claudia Martins Carneiro, Nilton Soares Camilo, Flaviane Francisco Hilário, Jason Guy Taylor

**Affiliations:** 1 Chemistry Department, ICEB, Federal University of Ouro Preto, Campus Universitário Morro do Cruzeiro, 35400-000 Ouro Preto, MG, Brazil; paulaelias23@yahoo.com.br (P.R.E.); silvagleicekelly@gmail.com (G.S.C.); vivifxavier@yahoo.com.br (V.F.X.); flavianehilario@iceb.ufop.br (F.F.H.); 2 Centro de Pesquisas René Rachou, A Fundação Oswaldo Cruz/FIOCRUZ, 30190-002 Belo Horizonte, MG, Brazil; policarpoasjunior@cpqrr.fiocruz.br (P.A.S.J.); romanha@cpqrr.fiocruz.br (A.J.R.); silvane@cpqrr.fiocruz.br (S.M.F.M.); 3 Immunopathology Laboratory, NUPEB, Federal University of Ouro Preto, Campus Universitário Morro do Cruzeiro, 35400-000 Ouro Preto-MG, Brazil; claudiamartinscarneiro@gmail.com; 4 Instituto de Química, Universidade Estadual de Campinas, C.P. 6154, CEP. 13084-971 Campinas, SP, Brazil; soares7@yahoo.com

**Keywords:** xylitan, xylitol, anhydropentitol, amastigote, trypomastigote, in vitro, chagas disease

## Abstract

A series of novel xylitan derivatives derived from xylitol were synthesized using operationally simple procedures. A xylitan acetonide was the key intermediate used to prepare benzoate, arylsulfonate esters and 1,2,3-triazole derivatives of xylitan. These compounds were evaluated for their in vitro anti-*Trypanosoma cruzi* activity against trypomastigote and amastigote forms of the parasite in *T. cruzi*-infected cell lineages. Benznidazole was used as positive control against *T. cruzi* and cytotoxicity was determined in mammalian L929 cells. The arylsulfonate xylitan derivative bearing a nitro group displayed the best activity of all the compounds tested, and was slightly more potent than the reference drug benznidazole. The importance of the isopropylidene ketal moiety was established and the greater lipophilicity of these compounds suggests enhancement in cell penetration.

## 1. Introduction

**Scheme 1:**
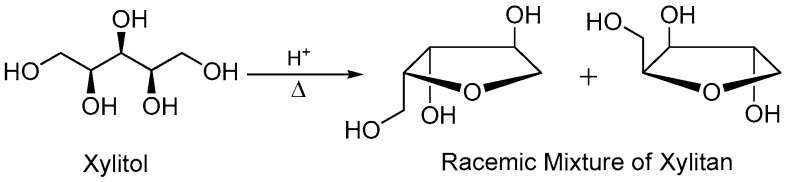
Synthesis of (±)-xylitan from xylitol.

The protozoan hemoflagellate *Trypanosoma cruzi* is the etiologic agent responsible for Chagas disease [1]. This disease is endemic to 21 Latin American countries [2,3] and almost half of those infected either have or will develop cardiomyopathy, digestive megasyndromes, or both [4]. This “neglected disease” is currently treated in Brazil with benznidazole, but unfortunately, this drug causes many significant side effects such as edema, fever, rash, agranulocytosis and neurotoxicity. Furthermore, the therapeutic effectiveness of benznidazole is dependent on the type of *T. cruzi* strain [5]. Nifurtimox has also been used to treat Chagas disease but due to the side effects of nifurtimox being more severe than benznidazole and its lower efficacy, the commercialization of nifurtimox was suspended in Brazil, Argentina, Chile and Uruguay in the 1980s. A number of studies focused on the synthesis and in vitro activity of biologically active compounds against *T. cruzi* have been reported in the literature. In vitro bioassays using the epimastigote form present in the midgut vector have been used as screening method for the evaluation of anti *T. cruzi* activity [6,7,8,9]. Intracellular amastigotes forms of *T. cruzi* have been targeted in recent studies due to the fact that they are present in the vertebrate host during the acute and chronic phases of the disease [10,11,12]. Compounds presenting promising in vitro activities against trypomastigote forms of the parasite have also been reported [13,14,15]. However, in vitro assays that allow the direct analysis of compounds on *T. cruzi*-infected cell lineages whilst monitoring simultaneously the effects on both amastigotes and trypomastigotes in just one system, are seldom employed in the discovery phase. This is particularly relevant for studies that seek to evaluate the parasite forms that are relevant to human infection. Following the guidelines suggested by the Fiocruz Program for Research and Technological Development on Chagas Disease Initiative, the whole cell-based screening methodology was utilized in the present study [16]. Soares and co-workers demonstrated that ribonucleosides were promising molecular scaffolds for the development of *T. cruzi* inhibitors [15] and their results motivated us to investigate anhydropentitol derivatives for therapy of parasitic diseases. Xylitol is a five-carbon sugar alcohol that has commercial uses in medicine and dietary applications as an alternative sweetener for diabetic persons. Xylitol can be converted to (±)-xylitan (also known as 1,4-anhydro-d/l-xylitol) via an acid catalyzed cyclisation reaction (Scheme 1) [17].

Synthetic efforts directed towards the preparation of xylitan derivatives have been scarce but some reports documenting the synthesis of these molecules have been described in the scientific literature [18,19,20,21,22]. Although many 1,4-anhydro-alditols have been extensively studied for their applications to treat infectious diseases, xylitan derivatives have been largely underexplored chemical frameworks for the development of bioactive compounds. In order to meet the deficit in new drug candidates for Chagas disease, we have prepared 22 xylitan derivatives and tested them against both amastigote and trypomastigote forms of *T. cruzi* in vitro.

## 2. Materials and Methods

All commercial reagents were used as received. Arylazides were prepared from the corresponding anilines following literature procedures [23]. Anhydrous solvents were purchased from Sigma Aldrich (St. Louis, MO, USA). Flash column chromatography was performed using silica gel 200–400 Mesh. TLC analyses were performed using silica gel plates, using ultraviolet light (254 nm), phosphomolybdic acid or vanillin solution for visualization. Melting points are uncorrected and were recorded on a Buchi B-540 apparatus (Zurich, Switzerland). For NMR data, the chemical shifts are reported in δ (ppm) referenced to residual solvent protons and ^13^C signals in deuterated chloroform. Coupling constants (*J*) are expressed in hertz (*Hz*). Infrared spectra were obtained on a Thermo Scientific Nicolet 380 FT-IR apparatus (600–4000 cm^−1^, Nicolet Instrument Corp., Madison, WI, USA) using attenuated total reflection (ATR). Mass spectra were obtained by GC-MS, Shimadzu QP-2010 Plus model (Shimadzu, Kyoto, Japan). SMILES notations of the xylitan derivatives were inputted into the online Molinspiration software (software version v2015.01) and only bioactive compounds were subjected to molecular properties prediction by Molinspiration software [24].

### 2.1. Synthesis of 3,5-O-Isopropylidene-1,4-xylitan *(**2**)*

To a round bottom flask (1000 mL) equipped with stir bar was added xylitan (100 g), anhydrous copper sulfate (100 g) and dry acetone (500 mL) at room temperature under an atmosphere of nitrogen. Concentrated sulfuric acid (0.3 mL) was added to the heterogeneous mixture and the system was kept under stirring at room temperature for 12 h. After this period, the reaction mixture was filtered through a sintered glass funnel. The organic phase was neutralized with an excess of calcium hydroxide, and filtered again on a sintered glass funnel. The mixture was then concentrated in vacuo to yield a viscous liquid. Purification was performed by column chromatography on flash silica gel eluting with ethyl acetate to afford xylitan-acetonide (±)-**2** (80%) as a white solid, m.p. 68–70 °C. ^1^H-NMR (250 MHz, CDCl_3_): δ 4.17–4.23 (m, 3H), 3.88–4.07 (m, 3H), 3.72 (d, *J* = 8.3 Hz, 1H), 2.96 (bs, OH), 1.41 (s, 3H), 1.33 (s, 3H); ^13^C-NMR (62.5 MHz, CDCl_3_): δ 97.4, 76.6, 75.4, 74.4, 72.0, 60.6, 28.5, 19.3.

### 2.2. Synthesis of (±)-3,5-O-Phenylboronate-1,4-xylitan *(**3**)*

In a round bottom flask equipped with stir bar and reflux condenser, xylitol (20 g; 131 mmol) and an aqueous solution of sulphuric acid (5.60 mL, 5% *v/v*) were added. The mixture was heated at 135 °C with stirring for 45 min. Upon cooling, xylitan was obtained as a viscous pale yellow liquid (16.40 g; 82%) and used in the next step without further purification. In a two necked round bottom flask equipped with stir bar, a Dean-Stark apparatus, a Liebig condenser and an addition funnel with pressure equalizing side arm, phenylboronic acid (0.874 g; 7.16 mmol) and 80 mL of benzene were added. A solution of xylitan **1** (0.800 g; 5.97 mmol) in 20 mL of methanol was added dropwise by addition funnel at 80 °C. Upon complete addition of xylitan, the temperature was raised to 100 °C and allowed to stir for 1 h. Finally, the reaction was allowed to cool to room temperature, the excess of benzene was removed under reduced pressure and the resulting liquid residue was triturated with a mixture of ethyl acetate and hexane (1:4) to afford compound (±)-**3** as a white solid in 47% yield. m.p. 103–104 °C; *R*_f_ = 0.48 (ethyl acetate/hexane, 2:3); IR (cm^−1^): 3441, 1595, 1448, 1310; ^1^H-NMR (500 MHz, CDCl_3_): δ 1.91 (brs, 1H), 3.80 (d, 1H, *J* = 10 Hz), 4.22–4.51 (m, 6H), 7.34 (t, 1H, *J* = 7.5 Hz), 7.43 (t, 2H, *J* = 7.5 Hz), 7.78 (d, 2H, *J* = 10 Hz); ^13^C-NMR (125 MHz, CDCl_3_): δ 61.2, 74.5, 74.6, 77.0, 77.4, 127.5, 130.9, 133.8, 134.4; EI *m*/*z* (%): 57 (40%), 149 (100%).

### 2.3. Synthesis of Benzoate Esters *(±)-**4a**–**g***

A general procedure is as follows: xylitan acetonide (±)-**2** (3 mmol) and a benzoyl chloride (3 mmol) or phthalic anhydride (3 mmol) were stirred in CH_2_Cl_2_ (5 mL) and pyridine (2 mL) at 0 °C for 0.5 h and then allowed to warm to room temperature. Stirring was continued for a further 16 h at room temperature. Upon completion, the reaction mixture was taken up into a separatory funnel containing a mixture of CH_2_Cl_2_ (15 mL) and a saturated aqueous solution of sodium bicarbonate (20 mL). The organic layer was washed with brine, dried over anhydrous Na_2_SO_4_, filtered, concentrated in vacuo and the crude product was purified by flash chromatography.

*(±)-2-Benzoyl-3,5-O-isopropylidene-1,4-xylitan* (**4a**): Product obtained as a white solid in 58% yield. m.p. 80–82 °C; *R*_f_ = 0.15 (ethyl acetate/hexane 2:8); IR (cm^−1^): 1725, 1449, 1263, 1174, 1102, 924, 782, 710; ^1^H-NMR (300 MHz, CDCl_3_): δ 1.41 (s, 3H), 1.46 (s, 3H), 3.96–4.48 (m, 6H), 5.39 (d, 1H, *J* = 3 Hz), 7.43 (t, 2H, *J* = 7.5 Hz), 7.57 (t, 1H, *J* = 7.5 Hz), 8.01 (d, 2H, *J* = 6 Hz); ^13^C-NMR (75 MHz, CDCl_3_): δ 19.4, 28.7, 60.6, 72.3, 72.6, 73.5, 79.3, 97.8, 129.6, 129.8, 133.5, 165.5; EI *m*/*z* (%): 177 (30%), 105 (100%), 77 (40%), 69 (50%).

*(±)-2-(4-Methoxybenzoyl)-3,5-O-isopropylidene-1,4-xylitan* (**4b**): Product obtained as a white solid in 25% yield. m.p. 102–104 °C; *R*_f_ = 0.48 (ethyl acetate/hexanes 2:8); IR (cm^−1^): 1699, 1592, 1449, 862, 756; ^1^H-NMR (300 MHz, CDCl_3_): δ 1.33 (s, 3H), 1.38 (s, 3H), 3.76–4.37 (m, 7H), 6.82 (d, 2H, *J* = 9 Hz), 7.87 (d, 2H, *J* = 9 Hz); ^13^C-NMR (75 MHz, CDCl_3_): δ 19.1, 28.5, 55.3, 60.3, 72.0, 72.4, 73.3, 78.8, 97.5, 113.5, 121.7, 131.6, 163.6, 165.0; EI *m*/*z* (%): 207 (30%), 135 (100%), 101 (60%).

*(±)-2-(3,4,5-Trimethoxybenzoyl)-3,5-O-isopropylidene-1,4-xylitan* (**4c**): Product obtained as a white solid in 12% yield. m.p. 120–122 °C; *R*_f_ = 0.45 (ethyl acetate/hexanes 2:8); IR (cm^−1^): 2989, 2945, 1699, 1592, 1449, 1227, 995, 862, 756; ^1^H-NMR (300 MHz, CDCl_3_): δ 1.44 (s, 3H), 1.49 (s, 3H), 3.92–4.51 (m, 15H), 5.40 (s, 1H), 7.29 (s, 2H); ^13^C-NMR (75 MHz, CDCl_3_): δ 19.5, 28.7, 56.4, 60.6, 61.1, 72.3, 72.7, 73.5, 79.5, 97.9, 107.1, 124.5, 142.8, 153.1, 165.3; EI *m*/*z* (%): 167 (25%), 95 (100%), 68 (60%).

*(±)-2-(3-Nitrobenzoyl)-3,5-O-isopropylidene-1,4-xylitan* (**4d**): Product obtained as a white solid in 63% yield. m.p. 110–111 °C; *R*_f_ = 0.30 (ethyl acetate/hexane 2:8); IR (cm^−1^): 2882, 1716, 1610, 1520, 969, 827, 719; ^1^H-NMR (300 MHz, CDCl_3_): δ 1.40 (s, 3H), 1.46 (s, 3H), 3.96–4.48 (m, 6H), 5.40 (d, 1H, *J* = 3 Hz), 7.66 (t, 1H, *J* = 9 Hz), 8.33 (d, 1H, *J* = 9 Hz), 8.41 (d, 1H, *J* = 9 Hz), 8.78 (s, 1H); ^13^C-NMR (75 MHz, CDCl_3_): δ 19.4, 28.6, 60.5, 72.1, 72.7, 73.4, 80.3, 97.9, 124.6, 127.9, 129.9, 131.3, 135.4, 148.4, 163.5; EI *m*/*z* (%): 104 (30%), 81 (100%).

*(±)-2-(4-Chlorobenzoyl)-3,5-O-isopropylidene-1,4-xylitan* (**4e**): Product obtained as a white solid in 54% yield. m.p. 198–199 °C; *R*_f_ = 0.48 (ethyl acetate/hexane 2:8); IR (cm^−1^): 1681, 1583, 1414, 916, 844, 800, 765, 684; ^1^H-NMR (300 MHz, CDCl_3_): δ 1.25 (s, 3H), 1.41 (s, 3H), 3.95–5.38 (m, 7H), 7.41 (d, 2H, *J* = 9 Hz), 7.94 (d, 2H, *J* = 9 Hz); ^13^C-NMR (75 MHz, CDCl_3_): δ 19.4, 28.7, 60.6, 72.3, 72.6, 73.5, 79.3, 97.8, 113.6, 121.7, 131.7, 163.6, 165.1; EI *m*/*z* (%): 313 (1%), 91 (100%), 68 (90%).

*(±)-2-(4-Methylbenzoyl)-3,5-O-isopropylidene-1,4-xylitan* (**4f**): Product obtained as a white solid in 55% yield. m.p. 85–87 °C; *R*_f_ = 0.25 (ethyl acetate/hexane 2:8); IR (cm^−1^): 2971, 1708, 1111, 969, 827, 746, 666; ^1^H-NMR (300 MHz, CDCl_3_): δ 1.41 (s, 3H), 1.46 (s, 3H), 2.40 (s, 3H), 3.95–4.48 (m, 6H), 5.37 (d, 1H, *J* = 6 Hz), 7.23 (d, 2H, *J* = 9 Hz), 7.90 (d, 2H, *J* = 9 Hz); ^13^C-NMR (75 MHz, CDCl_3_): δ 19.8, 22.2, 29.2, 61.0, 72.8, 73.1, 73.1, 79.6, 98.2, 127.3, 129.7, 130.2, 144.7, 166.1; EI *m*/*z* (%): 119 (100%), 91 (30%), 81 (30%), 69 (40%).

*(±)-2-(2-Carboxybenzoyl)-3,5-O-isopropylidene-1,4-xylitan* (**4g**): Product obtained as a pale yellow liquid in 35% yield. *R*_f_ = 0.46 (ethyl acetate/hexane 2:8); ^1^H-NMR (300 MHz, CDCl_3_): δ 1.40 (s, 3H), 1.45 (s, 3H), 3.77–4.10 (m, 6H), 4.40–4.58 (m, 1H), 5.37 (d, 1H, *J* = 3Hz), 7.55–7.58 (m, 2H), 7.70–7.77 (m, 2H); ^13^C-NMR (75 MHz, CDCl_3_): δ 19.5, 28.7, 60.6, 71.8, 72.6, 73.2, 80.1, 97.8, 129.2, 131.6, 166.4, 167.0. EI *m*/*z* (%): 69 (80%), 81 (70%), 98 (30%), 149 (100%), 211 (40%), 282 (42%).

### 2.4. Synthesis of Arylsulfonate Esters *(±)-**5a**–**g***

A general procedure is as follows: xylitan acetonide (±)-**2** (5 mmol), the appropriate arylsulfonyl chloride (10 mmol), 4-Dimethylaminopyridine (DMAP) (5 mol %) and CH_2_Cl_2_ (10 mL) were stirred together in round bottom flask at room temperature for 12 h. Next, the reaction mixture was taken up into a separatory funnel containing a mixture of CH_2_Cl_2_ (15 mL) and a saturated aqueous solution of sodium bicarbonate (20 mL). The organic layer was washed with brine, dried over anhydrous Na_2_SO_4_, filtered, concentrated in vacuo and the crude product was purified by flash chromatography.

*(±)-2-Benzenesulfonyl-3,5-O-isopropylidene-1,4-xylitan* (**5a**): Product obtained as a white solid in 74% yield. m.p. 62–64 °C; *R*_f_ = 0.19 (ethyl acetate/hexane 6:4); IR (cm^−1^): 1440, 1356, 910, 826, 756, 684; ^1^H-NMR (300 MHz, CDCl_3_): δ 1.31 (s, 3H), 1.35 (s, 3H), 3.76–4.04 (m, 4H), 4.20 (dd, 1H, *J* = 9 Hz, *J* = 3 Hz), 4.33 (s, 1H); 4.86 (d, 1H, *J* = 6 Hz), 7.54–7.70 (m, 3H), 7.90 (d, 2H, *J* = 9 Hz); ^13^C-NMR (75 MHz, CDCl_3_): δ 19.3, 28.6, 60.3, 71.6, 72.1, 73.5, 84.6, 97.8, 127.9, 129.5, 134.3, 136.4; EI *m*/*z* (%): 299 (20%), 141 (30%), 81 (80%), 69 (100%), 59 (40%).

*(±)-2-(4-Methylbenzenesulfonyl)-3,5-O-isopropylidene-1,4-xylitan* (**5b**): Product obtained as a white solid in 73% yield. m.p. 82–85 °C; *R*_f_ = 0.48 (ethyl acetate/hexanes 6:4); IR (cm^−1^): 3004, 2912, 1592, 1355, 1188, 1099, 1050, 894, 809, 778, 667; ^1^H-NMR (300 MHz, CDCl_3_): 1.33 (s, 3H), 1.38 (s, 3H), 2.45 (s, 3H), 3.76 4.05 (m, 4H), 4.20 (dd, 1H, *J* = 7.5 Hz, *J* = 4.5 Hz), 4.35 (s, 1H), 4.84 (d, 1H, *J* = 3 Hz), 7.36 (d, 2H, *J* = 6 Hz), 7.79 (d, 2H, *J* = 6 Hz); ^13^C-NMR (75 MHz, CDCl_3_): 19.3, 21.8, 28.6, 60.4, 71.7, 72.2, 73.6, 84.5, 97.9, 128.0, 130.2, 133.4, 145.5; EI *m*/*z* (%): 312 (20%), 155 (60%), 91 (70%), 81 (60%), 68 (100%).

*(±)-2-(4-Chlorobenzenesulfonyl)-3,5-O-isopropylidene-1,4-xylitan* (**5c**): Product obtained as a white solid in 47% yield. m.p. 74–75 °C; *R*_f_ = 0.37 (ethyl acetate/hexane 2:8); IR (cm^−1^): 2909, 1699, 1574, 1485, 1360, 889, 800; ^1^H-NMR (300 MHz, CDCl_3_): δ 1.33 (s, 3H), 1.39 (s, 3H), 3.78–4.05 (m, 4H), 4.24 (dd, 1H, *J* = 7.5 Hz, *J* = 3 Hz), 4.36 (s, 1H), 4.86 (d, 1H, *J* = 3 Hz), 7.55 (d, 2H, *J* = 6 Hz), 7.85 (d, 2H, *J* = 6 Hz); ^13^C-NMR (75 MHz, CDCl_3_): δ 19.3, 28.6, 60.3, 71.6, 72.2, 73.6, 84.9, 97.9, 129.4, 129.9, 134.8, 141.1; EI *m*/*z* (%): 333 (20%), 175 (30%), 111 (30%), 69 (100%), 59 (60%).

*(±)-2-(4-Fluorobenzenesulfonyl)-3,5-O-isopropylidene-1,4-xylitan* (**5d**): Product obtained as a pale yellow liquid in 67% yield. *R*_f_ = 0.24 (ethyl acetate/hexanes 3:7); IR (cm^−1^): 2918, 1699, 1592, 1502, 1369, 1114, 1102, 960, 898, 844; ^1^H-NMR (300 MHz, CDCl_3_): δ 1.27 (s, 3H), 1.33 (s, 3H), 3.73–4.00 (m, 4H), 4.18 (dd, 1H, *J* = 7.5 Hz, *J* = 4.5 Hz), 4.31 (s, 1H), 4.82 (d, 1H, *J* = 3 Hz), 7.21(t, 2H, *J* = 7.5 Hz), 7.87–7.92 (m, 2H); ^13^C-NMR (75 MHz, CDCl_3_): δ 19.2, 28.4, 60.1, 71.4, 72.0, 73.4, 84.7, 97.7, 116.8 (d, C-F, *J* = 23.2 Hz), 130.7 (d, C-F, *J* = 9.7 Hz), 132.3 (d, C-F, *J* = 3Hz), 165.9 (d, C-F, *J* = 255.7 Hz); EI *m*/z (%): 317 (20%), 159 (30%), 95 (40%), 69 (100%).

*(±)-2-(4-Methoxybenzenesulfonyl)-3,5-O-isopropylidene-1,4-xylitan* (**5e**): Product obtained as a pale yellow liquid in 19% yield. *R*_f_ = 0.45 (ethyl acetate/hexane 3:7); IR (cm^−1^): 1601, 1502, 1369, 898, 809; ^1^H-NMR (300 MHz, CDCl_3_): δ 1.31 (s, 3H), 1.36 (s, 3H), 3.75–4.33 (m, 10H), 4.80 (s, 1H), 7.00 (d, 2H, *J* = 6 Hz), 7.82 (d, 2H, *J* = 6 Hz); ^13^C-NMR (75 MHz, CDCl_3_): δ 19.7, 29.0, 56.3, 60.8 72.1, 72.5, 74.0, 84.7, 98.2, 115.1, 128.0, 130.6, 164.6; EI *m*/*z* (%): 328 (50%), 188 (70%), 171 (100%), 107(30%), 81 (70%), 59 (60%).

*(±)-2-(4-Nitrobenzenesulfonyl)-3,5-O-isopropylidene-1,4-xylitan* (**5f**): Product obtained as a white solid in 60% yield. m.p. 116–118 °C; *R*_f_ = 0.52 (ethyl acetate/hexanes 2:8); IR (cm^−1^): 3117, 1602, 1529, 1356, 1314, 1193, 1099, 1039, 894, 732, 686; ^1^H-NMR (300 MHz, CDCl_3_): δ 1.34 (s, 3H), 1.40 (s, 3H), 3.82–4.07 (m, 4H), 4.27 (dd, 1H, *J* = 3 Hz, *J* = 6 Hz), 4.40 (d, 1H, *J* = 3 Hz), 4.95 (d, 1H, *J* = 3 Hz), 8.12 (d, 2H, *J* = 9 Hz), 8.42 (d, 2H, *J* = 9 Hz); ^13^C-NMR (75 MHz, CDCl_3_): δ 19.5, 28.6, 60.3, 71.6, 72.3, 73.6, 85.7, 98.1, 124.8, 129.3, 142.1, 151.1; EI *m*/*z* (%): 344 (10%), 69 (100%).

*(±)-2-(8-Quinolinesulfonyl)-3,5-O-isopropylidene-1,4-xylitan* (**5g**): Product obtained as a white solid in 32% yield. m.p. 129–131 °C; *R*_f_ = 0.24 (ethyl acetate/hexanes 4:6); IR (cm^−1^): 2997, 2934, 2904, 1561, 1499, 1455, 1362, 1278, 1176, 1088, 910, 840, 797, 743, 668; ^1^H-NMR (300 MHz, CDCl_3_): δ 1.34 (s, 3H), 1.36 (s, 3H), 3.84–4.04 (m, 4H), 4.28 (d, 1H, *J* = 3 Hz), 4.46 (s, 1H), 5.52 (s, 1H), 7.54–7.67 (m, 2H), 8.13 (d, 1H, *J* = 4, 5 Hz), 8.27 (d, 1H, *J* = 6 Hz), 8.50 (d, 1H, *J* = 6 Hz), 9.08 (s, 1H); ^13^C-NMR (75 MHz, CDCl_3_): δ 19.7, 29.1, 60.9, 72.4, 72.6, 74.3, 86.6, 98.2, 123.0, 125.9, 129.5, 133.7, 134.7, 135.5, 137.1, 144.2, 152.3; EI *m*/*z* (%): 210 (50%), 192 (20%), 129 (100%), 101 (20%), 81 (20%), 59 (20%).

### 2.5. Synthesis of (±)-2-Benzyl-3,5-O-isopropylidene-1,4-xylitan *(**6**)*

Xylitan acetonide (±)-**2** (5 mmol) dissolved in anhydrous THF (15 mL) was added to sodium hydride (6 mmol) at 0 °C under an inert atmosphere of nitrogen. The mixture was stirred for 10 min and benzyl bromide (6 mmol) was added slowly to the mixture, allowed to warm to room temperature and left to stir for a further 24 h. Distilled water (15 mL) was added to quench the reaction and the organic product was extracted from the aqueous phase with ethyl acetate (2 × 20 mL) in a separatory funnel. The organic layer was washed with brine, dried over anhydrous Na_2_SO_4_, filtered, concentrated in vacuo and the crude product was purified by flash chromatography to afford a pale yellow liquid in 15% yield. *R*_f_ = 0.31 (ethyl acetate/hexane 2:8); ^1^H-NMR (300 MHz, CDCl_3_): δ 1.37 (s, 3H), 1.44 (s, 3H), 3.86–4.33 (m, 7H), 4.57 (s, 2H), 7.33 (s, 5H); ^13^C-NMR (75 MHz, CDCl_3_): δ 19.4, 28.8, 60.8, 71.7, 72.1, 72.4, 73.5, 84.2, 97.5, 127.7, 127.1, 128.6; EI *m*/*z* (%): 264 (10%), 249 (60%), 157 (30%), 91 (100%), 77 (10%).

### 2.6. Synthesis of (±)-2-(4-Methylbenzenosulfonyl)-1,4-xylitan *(**7**)*

To a round bottom flask equipped with stir bar was added (±)-**5b** (0.3 mmol), CH_2_Cl_2_ (5 mL) and TFA (0.9 mL). The reaction was stirred for 1 h at room temperature and after this time, the solvent was removed under reduced pressure and the resultant liquid residue was transferred directly to a column and purified by flash chromatography. Upon purification, compound (±)-**7** was obtained as a transparent liquid in 15% yield. *R*_f_ = 0.55 (ethyl acetate/hexane 3:7); ^1^H-NMR (300 MHz, CDCl_3_): δ 2.46 (s, 3H), 3.83–4.23 (m, 5H), 4.41 (s, 1H), 4.81 (s, 1H), 7.36 (d, 2H, *J* = 9 Hz), 7.79 (d, 2H, *J* = 9 Hz); ^13^C-NMR (75 MHz, CDCl_3_): δ 21.4, 61.2, 70.6, 78.6, 85.1, 109.7, 127.6, 129.8, 132.8, 145.1; EI *m*/*z* (%): 91 (100%), 81 (80%), 68 (80%).

### 2.7. Synthesis of (±)-3,5-Isopropylidene-1,4-anidro-2-deoxy-pent-1-enitol *(**9**)*

Xylitan-acetonide (±)-**2** (2.4 g, 13.9 mmol), anhydrous CH_2_Cl_2_ (20 mL) and triethylamine (5.8 mL, 41.7 mmol), were stirred in a round bottom flask under an atmosphere of nitrogen at −10 °C. Next, methanesulfonyl chloride (2.2 mL, 27.7 mmol), was added dropwise by addition funnel and the mixture was allowed to stir at room temperature for 2 h. The reaction was quenched by the addition of distilled water (20 mL) and then taken up into a separatory funnel. The organic layer was washed with HCl (1 M, 10 mL), saturated solution of Na_2_CO_3_ (20 mL) and finally the organic layer was evaporated under reduced pressure to afford (±)-**8** as a light brown solid in 92% yield. An analytical sample of (±)-**8** was obtained by recrystallization with EtOAc/Hex. White solid, m.p. 93–94 °C. *R*_f_ = 0.75 (ethyl acetate/hexane 3:7); IR (cm^−1^): 2999, 2934, 1349, 1179, 1095, 1042; ^1^H-NMR (400 MHz, CDCl_3_): δ 1.35 (s, 3H), 1.43 (s, 3H), 3.05 (s, 3H), 3.88–4.09 (m, 4H), 4.35 (dd, 1H, *J* = 12 Hz, *J* = 8 Hz), 4.48 (s, 1H), 5.03 (d, 1H, *J* = 4 Hz); ^13^C-NMR (100 MHz, CDCl_3_): δ 19.3, 28.5, 38.5, 60.3, 71.8, 72.1, 73.5, 83.7, 97.8; EI *m*/*z* (%): 81 (90%), 69 (100%), 59 (60%), 57 (40%). Compound (±)-**8** (4 mmol) was stirred in 1,8-Diazabicyclo[5.4.0]undec-7-ene (DBU) (10 mL) at 150 °C for 45 min then cooled to room temperature and extracted with water (30 mL) and ethyl acetate (40 mL) in a seperatory funnel. The organic layer was washed with brine, dried over anhydrous Na_2_SO_4_, filtered, concentrated in vacuo and the crude product was purified by flash chromatography to provide a viscous pale yellow liquid in 56% yield *R*_f_ = 0.36 (Hexane/EtOAc, 5:1) after visualization by vanillin; ^1^H-NMR (250 MHz, CDCl_3_): δ 6.67 (d, *J* = 2.8 Hz, 1H), 5.17 (t, *J* = 2.5 Hz, 1H), 4.93 (dd, *J* = 2.8, 6.5 Hz, 1H), 4.37 (dd, *J* = 6.0, 12.5 Hz, 1H), 4.06 (dd, *J* = 6.0, 12.0 Hz, 1H), 3.87 (dd, *J* = 6.5, 12.0 Hz, 1H), 1.41 (s, 3H), 1.38 (s, 3H)); EI *m*/*z* (%) = 81 (100%), 68 (35%).

### 2.8. Synthesis of (±)-2-Propargyl-3,5-O-isopropylidene-1,4-xylitan *(**10**)*

To a round bottom flask equipped with stir bar and 4 Å molecular sieves (1 g), was added xylitan acetonide (±)-**2** (3.2 mmol), sodium tert-butoxide (6.3 mmol) and acetonitrile (35 mL). The solution was cooled to 0 °C and a solution of propargyl chloride (3.2 mmol in 7 mL of CH_3_CN) was added dropwise by addition funnel. Upon addition of propargyl chloride, the solution was allowed to warm to room temperature and stirred for a further 12 h. Next, the solution was filtered to remove the molecular sieves and the filtrate was taken up into a separatory funnel containing distilled water (20 mL). The product was extracted from the aqueous phase with ethyl acetate (3× 20 mL) and the organic phase extractions were combined, washed with brine, dried over anhydrous Na_2_SO_4_, filtered and concentrated in vacuo. The crude product was purified by flash chromatography to furnish a (±)-**10** as a transparent liquid in 86% yield. *R*_f_ = 0.36 (ethyl acetate/hexane 1:9); ^1^H-NMR (300 MHz, CDCl_3_): δ 1.34 (s, 3H), 1.42 (s, 3H), 2.45 (s, 1H), 3.80–4.31 (m, 8H); ^13^C-NMR (75 MHz, CDCl_3_): δ 19.3, 28.7, 56.9, 60.6, 71.8, 72.3, 73.2, 75.1, 79.3, 83.8, 97.6; EI *m*/*z* (%): 197 (30%), 101 (65%), 82 (100%).

### 2.9. Synthesis of Triazoles ***11a**–**d***

To a one necked round bottom flask equipped with stir bar was added freshly prepared arylazide (2.7 mmol), a solution of CuSO_4_·5H_2_O (0.44 mmol in 3 mL of H_2_O) and a solution of sodium ascorbate (0.56 mmol in 3 mL of H_2_O). The mixture was stirred vigorously and a solution of (±)-**10** (1.4 mmol in 6 mL of CH_2_Cl_2_) was added dropwise and allowed to stir at room temperature for 24 h. The reaction mixture was transferred to a separatory funnel and extracted with CH_2_Cl_2_ (2 × 25 mL). The organic layer was washed with brine, dried over anhydrous Na_2_SO_4_, filtered, concentrated in vacuo and the crude product was purified by flash chromatography.

*(±)-2-[(1’-(4-Methoxyphenyl)-2-O-metil-1H-1,2,3-triazol]-3,5-O-isopropylidene-1,4-xylitan* (**11a**): Product obtained as a light brown solid in 85% yield. m.p. 106–108 °C; *R*_f_ = 0.29 (ethyl acetate/hexane 2:8); IR (cm^−1^): 2936, 1494, 1201, 1102, 1031, 969, 836, 746; ^1^H-NMR (300 MHz, CDCl_3_): δ 1.35 (s, 3H), 1.43 (s, 3H), 3.84–4.37 (m, 7H), 4.74 (s, 2H), 6.99 (d, 2H, *J* = 9 Hz), 7.58 (d, 2H, *J* = 9 Hz), 7.88 (s, 1H); ^13^C-NMR (75 MHz, CDCl_3_): δ 19.4, 28.8, 55.7, 60.7, 63.1, 72.0, 72.3, 73.2, 84.4, 97.6, 114.8, 121.1, 122.3, 130.4, 144.1 159.9; EI *m*/*z* (%): 361 (1%), 167 (25%), 149 (100%), 57 (60%).

*(±)-2-[(1’-(4-Chlorophenyl)-2-O-metil-1H-1,2,3-triazol]-3,5-O-isopropylidene-1,4-xylitan* (**11b**): Product obtained as a white solid in 41% yield. m.p. 134–136 °C; *R*_f_ = 0.52 (ethyl acetate/hexane 1:9); IR (cm^−1^): 3007, 2909, 1520, 1449, 1272, 1209, 1111, 1040, 978, 827; ^1^H-NMR (300 MHz, CDCl_3_): δ 1.37 (s, 3H), 1.45 (s, 3H), 3.86–4.38 (m, 7H), 4.77 (s, 2H), 7.48–7.52 (m, 2H), 7.66–7.70 (m, 2H), 7.95 (s, 1H); ^13^C-NMR (75 MHz, CDCl_3_): *δ* 19.5, 28.7, 60.7, 63.2; 72.0, 72.5, 73.3, 83.9, 84.7, 97.5, 120.7, 121.7, 129.1, 134.7, 135.4; EI *m*/*z* (%): 167 (30%), 149 (80%), 71 (60%), 57 (100%).

*(±)-2-[(1’-(2-Acylphenyl)-2-O-metil-1H-1,2,3-triazol]-3,5-O-isopropylidene-1,4-xylitan* (**11c**): Product obtained as a white solid in 29% yield. m.p. 135–136 °C; *R*_f_ = 0.29 (ethyl acetate/hexane 2:8); IR (cm^−1^): 2900, 1502, 1440, 960, 809, 756; ^1^H-NMR (400 MHz, CDCl_3_): δ 1.37 (s, 3H), 1.45 (s, 3H), 2.65 (s, 3H), 3.75–4.39 (m, 7H), 4.78 (s, 2H), 7.86 (d, 2H, *J* = 8 Hz), 8.05 (s, 1H), 8.12 (d, 2H, *J* = 8 Hz); ^13^C-NMR (100 MHz, CDCl_3_): δ 19.9, 27.1, 29.1, 61.0, 63.5, 72.3, 72.8, 73.7, 85.0, 98.0, 120.5, 121.0, 130.6, 137.4, 140.4, 146.3, 196.1; EI *m*/*z* (%): 207 (50%), 73 (100%).

*(±)-2-[(1’-(3,4-Dimethylphenyl)-2-O-metil-1H-1,2,3-triazol]-3,5-O-isopropylidene-1,4-xylitan* (**11d**): Product obtained as a white solid in 34% yield. m.p. 110–111 °C; *R*_f_ = 0.13 (ethyl acetate/hexane 2:8); IR (cm^−1^): 2936, 1601, 1440, 1378, 1201, 1066, 960, 827, 756; ^1^H-NMR (400 MHz, DMSO): δ 1.23 (s, 3H), 1.39 (s, 3H), 2.28 (s, 3H), 2.31 (s, 3H), 3.68–4.07 (m, 6H), 4.39 (m, 1H), 4.68 (s, 2H); 7.33–7.41 (m, 2H), 7.59 (d, 1H, *J* = 4 Hz), 8.74 (s, 1H); ^13^C-NMR (100 MHz, DMSO): δ 19.1, 19.6, 28.8, 60.1, 62.1, 71.4, 71.9, 72.7, 83.7, 96.9, 117.5, 121.1, 122.3, 130.7, 134.7, 137.2, 138.3, 144.8; EI *m*/*z* (%): 202 (40%), 158 (100%), 145 (51%), 105 (70%).

### 2.10. Anti-Trypanosoma cruzi Activity Assay

The in vitro anti-*T. cruzi* activity was evaluated on L929 cells (mouse fibroblasts) infected with Tulahuen strain of the parasite expressing the *Escherichia coli* β-galactosidase as reporter gene according to the method described previously [16]. Briefly, for the bioassay, 4000 L929 cells were added to each well of a 96-well microtiter plate. After an overnight incubation, 40,000 trypomastigotes were added to the cells and incubated for 2 h. Then the medium containing extracellular parasites was replaced with 200 μL of fresh medium and the plate was incubated for an additional 48 h to establish the infection. For IC_50_ determination, the cells were exposed to each synthesized compound at serial decreasing dilutions and the plate was incubated for 96 h. After this period, 50 μL of 500 μM chlorophenol red beta-d-galactopyranoside (CPRG) in 0.5% Nonidet P40 was added to each well, and the plate was incubated for 16–20 h, after which the absorbance at 570 nm was measured. Controls with uninfected cells, untreated infected cells, infected cells treated with benznidazole at 3.8 μM (positive control) or DMSO 1% were used. The results were expressed as the percentage of *T. cruzi* growth inhibition in compound-tested cells as compared to the infected cells and untreated cells. The IC_50_ values were calculated by linear interpolation. Quadruplicates were run in the same plate, and the experiments were repeated at least once.

## 3. Results and Discussion

### 3.1. Chemistry

**Scheme 2:**
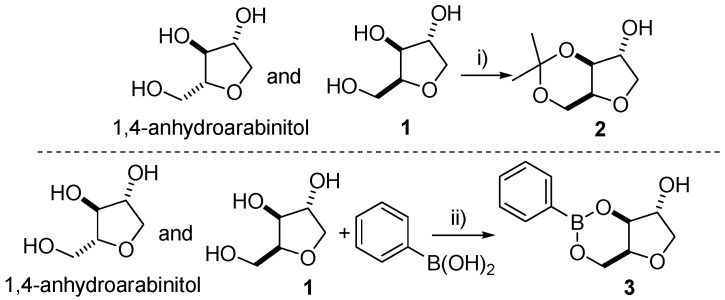
Synthetic route for the preparation of (±)-xylitan acetonide **2** and boronate ester **3**: (i) CuSO_4_, acetone, room temperature, 18 h, 75% yield; (ii) methanol–benzene, Dean-Stark, 1 h, reflux, 47%.

Heating xylitol in the presence of a strong mineral acid, sulfuric acid, with concomitant removal of water yields a mixture of (±)-xylitan and (±)-1,4-anhydroarabinitol in a ratio of 12:1, as determined by integration of the ^1^H-NMR signals. The key intermediate for the preparation of the target molecules was obtained by exploitation of the *cis* diol for the introduction of an isopropylidene ketal. (±)-Xylitan acetonide **2** was obtained in gram quantities by reacting (±)-**1** with acetone in the presence of CuSO_4_ at room temperature (Scheme 2). As expected, only (±)-xylitan containing two contiguous hydroxyls disposed in a *cis* configuration reacts to form (±)-**2**. This allows for easy separation of the desired product from the unreacted (±)-1,4-anhydroarabinitol by column chromatography. In a similar fashion, the boronate ester (±)-**3** was obtained by reacting (±)-xylitan **1** with phenylboronic acid in refluxing benzene. Intermediate (±)-**3** is a novel compound and could be potentially modified at the phenyl ring or substituted with other groups by employing substituted phenylboronic acids.

**Scheme 3:**
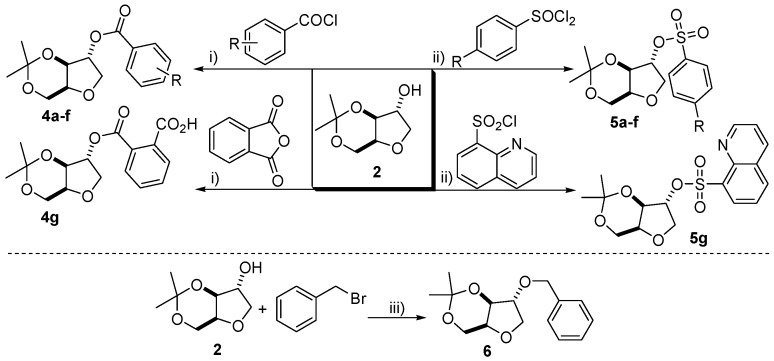
Synthetic route for the preparation of (±)-xylitan acetonide derivatives: (i) pyridine, CH_2_Cl_2_, rt, 16 h, see Table 1; (ii) pyridine, 4-Dimethylaminopyridine (DMAP) (5 mol %), CH_2_Cl_2_, rt, 16 h, see Table 1; (iii) NaH, THF, 0 °C–room temperature, 24 h, 15%.

Next, the free hydroxyl group of (±)-**2** was reacted with substituted benzoyl chlorides and arylsulfonyl chlorides to provide a range structurally related analogues, (±)-**4** and (±)-**5**, containing either electron donating or withdrawing groups (Scheme 3). Phthalic anhydride was reacted with (±)-**2** in pyridine at room temperature to afford (±)-**4g**. A sulfonyl quinoline derivative (±)-**5g** was also obtained in good yield using the standard procedure (Scheme 3). In order to discern the importance of the carbonyl ester for anti *T. cruzi* activity, compound (±)-**6**, the ether equivalent of (±)-**4a** was synthesized by alkylation of (±)-**2** with benzyl bromide.

**Scheme 4:**
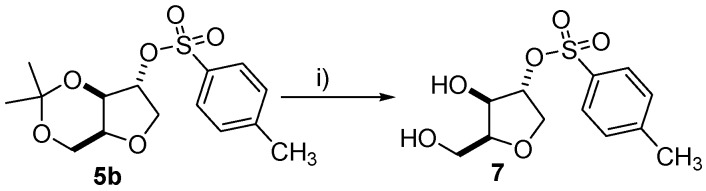
Deprotection of (±)-**5b**: (i) TFA, CH_2_Cl_2_, 0.5 h, room temperature, 15%.

In order to evaluate the importance of the isopropylidene ketal for anti *T. cruzi* activity, deprotection of (±)-**5b** was carried out using TFA to liberate the *cis* diol which furnished compound (±)-**7** (Scheme 4).

**Scheme 5:**
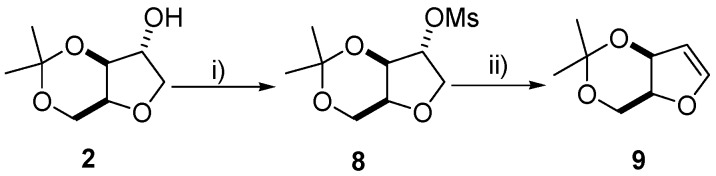
Synthesis of glycal (±)-**9**: (i) CH_3_SO_2_Cl, CH_2_Cl_2_, Et_3_N, 2 h, 0 °C, 96%; (ii) 1,8-Diazabicyclo[5.4.0]undec-7-ene (DBU), 0.5 h, 150 °C, 56%.

Xylitan acetonide (±)-**2** was mesylated smoothly to provide (±)-**8** in good yield (Scheme 5). Unfortunately, compound (±)-**8** was unreactive towards nucleophilic substitution with phenols, anilines and azides. Thus, all attempts at introducing other functionalities at the C-2 position with inversion of configuration were unsuccessful. In contrast, when (±)-**8** was treated with a sterically hindered base such as 1,8-Diazabicyclo[5.4.0]undec-7-ene (DBU) at elevated temperatures (150 °C), an elimination reaction ensued that provided the corresponding glycal (±)-**9** (Scheme 5), albeit, in only modest yield (56%).

**Scheme 6:**
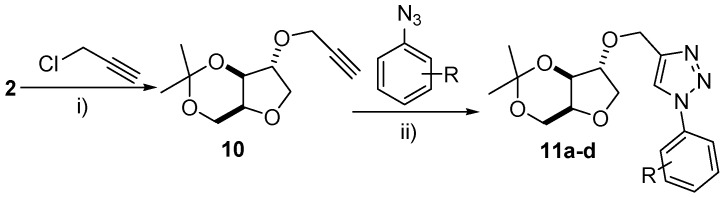
Synthesis of xylitan-triazole compounds (±)-**11a**–**d**: (i) *t*-BuONa, CH_3_CN, 0 °C, 12 h, 86%; (ii) CuSO_4_·5H_2_O, NaAsc, EtOH/H_2_O, room temperature, see Table 2.

Andrade and co-workers reported the biological evaluation of 1,2,3-triazole-based analogues of benznidazole [24] and their promising results inspired us to incorporate a pendant triazole moiety into xylitan acetonide (±)-**2**. To achieve this goal, the alkylation of (±)-**2** with propargyl chloride was carried out and the resulting ether (±)-**10** was subsequently coupled to aromatic azides via a cycloaddition reaction to provide the desired triazoles (±)-**11** in modest yields (Scheme 6).

Compounds (±)-**3**–**11** are novel and were characterized by mass spectrometry, FT-IR ^1^H- and ^13^C-NMR spectroscopy.

### 3.2. Biology

The antiprotozoal activity of compounds (±)-**2**–**11** were evaluated in vitro against trypomastigote and amastigote forms of *T. cruzi* using the method described by Romanha et al. [16]. Benznidazole was used as positive control against *T. cruzi* and cytotoxicity was determined in mammalian L929 cells (Table 3). Our reference compound provided an IC_50_ of 3.8 μM and gave a selectivity index (SI) of 625 and was used as a benchmark value for assessing the potency and selectivity of the xylitan derivatives. With these targets in mind, only those compounds that exhibited IC_50_ < 10 μM were further evaluated for their cytotoxicity. Many of the compounds described herein were inactive at the tested concentrations. Amongst the carboxylic acid esters (±)-**4a**–**g**, only (±)-**4e** which contains a chloro substituent at the *para* position, demonstrated significant trypanocidal activity, but was almost four times less potent than benznidazole. The arylsulfonate esters were in general more active than their carboxylic acid ester counterparts and compounds (±)-**5b**, (±)-**5d**, (±)-**5f** and (±)-**5g** displayed IC_50_ values in the range of 2.7 to 29.5 μM. Notably, compounds containing electron withdrawing groups demonstrated greatest activity amongst the compounds tested (NO_2_ > F > CH_3_). In order to evaluate the importance of the ketal group for trypanocidal activity, compound (±)-**5** was prepared and tested. The deprotected compound was essentially inactive unlike compound (±)-**5b** and so the decision was taken to maintain the isopropylidene ketal group. The calculated Log P of compound (±)-**7** was found to be 1.15 whereas in comparison, (±)-**5b** was 2.09. It is unclear at this stage if the isopropylidene ketal enhances potency or merely renders the xylitan derivatives more lipophilic and thus favouring greater penetration into the parasite. The stand out lead compound was xylitan derivative (±)-**5f** which contains a nitro group. This novel compound was slightly more active than the positive control and moderately selective (SI = 13). The physicochemical drug descriptors of the molecular properties for the synthesized compounds were calculated by Molinspiration software (Table 3). The partition coefficient (LogP: octanol/water partition coefficient) describes the equilibrium distribution between two liquid phases such as octanol and water and the total polar surface area (TPSA) is a measure of the extent of the molecules exposed polar area. Although there were no linear correlations between molecular hydrophobicity and bioactivity, all Log *P* values for the bioactive compounds were found to be less than 5 which satisfies Lipinski’s rule of five and suggests potentially good permeability across cell membranes. Interestingly, compound (±)-**5f** presented the highest TPSA value which is below the limit of 140 Å^2^ (Lipinski’s rule of five); based on the TPSA value alone, it would be expected to perform the poorest at permeating cell membranes. The presence of the nitro group may therefore be important for enhancing a protein-ligand interaction at a specific target that is responsible for its greater potency in vitro. Given the promising results described by the group of Andrade [25], we were surprised to find that triazoles (±)-**11a**–**d** were inactive against *T. cruzi*.

## 4. Conclusions

In conclusion, although the potency of many of the xylitan derivatives was less active than the positive control, potency was enhanced by the inclusion of halogens, nitro group or a quinoline moiety. In one case, the xylitan derivative (±)-**5f** was slightly more potent than benznidazole. The xylitan framework and other anhydropentitols may assist in crossing biologic membranes and in penetrating into the parasite.

## Figures and Tables

**Table 1:** Product yields for compounds **4a**–**g** and **5a**–**g**.

Compound	R	Yield (%)
(±)-**4a**	H	58
(±)-**4b**	4-OCH_3_	25
(±)-**4c**	3,4,5-(OCH_3_)_3_	12
(±)-**4d**	3-NO_2_	63
(±)-**4e**	4-Cl	54
(±)-**4f**	4-CH_3_	55
(±)-**4g**	2-CO_2_H	35
(±)-**5a**	H	74
(±)-**5b**	CH_3_	73
(±)-**5c**	Cl	47
(±)-**5d**	F	67
(±)-**5e**	OCH_3_	19
(±)-**5f**	NO_2_	60
(±)-**5g**	Quinoline	32

**Table 2:** Product yields for compounds **11a**–**d**.

Compound	R	Yield (%)	
**11a**	4-OCH_3_		85
**11b**	4-Cl		41
**11c**	4-COCH_3_		29
**11d**	3,4-(CH_3_)_2_		34

**Table 3:** In vitro trypanocidal activity, cytotoxicity and selectivity index (SI) of bioactive xylitan derivatives.

Compound	R	Trypanocide IC_50_ (μM)	Cytotoxicity CC_50_ (μM)	SI	Log P	TPSA (Å^2^)
(±)-**4e**	4-Cl	15.6 (± 2.2)	ND	-	3.22	54.01
(±)-**4g**	2-CO_2_H	324.0 (± 54)	ND	-	1.73	91.31
(±)-**5b**	4-CH_3_	16.4 (± 2.2)	ND	-	2.09	71.08
(±)-**5d**	4-F	6.6 (± 2.2)	13.3	2	1.80	71.08
(±)-**5f**	4-NO_2_	2.7 (± 2.2)	35.9	13	1.60	116.90
(±)-**5g**	Quinoline	29.2 (± 3.0)	ND	-	1.60	83.97
Benznidazole	-	3.8	2381	625	-	-

IC_50_: 50% inhibitory concentration. ND: not determined. CC_50_: 50% cytotoxic concentration. SI: selectivity index calculated from CC_50_/IC_50_. TPSA: total polar surface area.
